# Dietary supplementation with β-mannanase and probiotics as a strategy to improve laying hen performance and egg quality

**DOI:** 10.3389/fvets.2023.1229485

**Published:** 2023-12-05

**Authors:** Camila Lopes Carvalho, Ines Andretta, Gabriela Miotto Galli, Thais Bastos Stefanello, Nathalia de Oliveira Telesca Camargo, Ricardo Evandro Mendes, Giovanna Pelisser, Balasubramanian Balamuralikrishnan, Raquel Melchior, Marcos Kipper

**Affiliations:** ^1^Department of Animal Science, Faculdade de Agronomia, Universidade Federal do Rio Grande do Sul, Porto Alegre, Brazil; ^2^Department of Pathobiology, Pharmacology and Zoological Medicine, Faculty of Veterinary Medicine, Ghent University, Merelbeke, Belgium; ^3^Laboratory of Veterinary Pathology, Instituto Federal Catarinense, Concórdia, Brazil; ^4^Department of Food Science and Biotechnology, Sejong University, Seoul, Republic of Korea; ^5^Elanco Animal Health, São Paulo, Brazil

**Keywords:** additives, biochemical indicators, egg quality, feeding, gut health, nutrition

## Abstract

The objective of this study was to assess the impact of β-mannanase and probiotic on the performance, serum biochemistry, gut morphometric traits, and fresh egg quality of laying hens. A total of 120 cages, housing light-weight laying hens (36 weeks old), were randomly assigned to four different treatments. These treatments included a control group fed non-supplemented diets; diets supplemented with 300 g/ton of beta-mannanase; diets supplemented with 50 g/ton of probiotic; or diets containing both 300 g/ton of β-mannanase and 50 g/ton of probiotics. The trial spanned a duration of 26 weeks and was divided into three productive phases, each lasting 28 days. The inclusion of β-mannanase resulted in a significant improvement in the laying rate by 11% (*p* < 0.05) compared to the control treatment. Similarly, the addition of probiotics also enhanced the laying rate by 7% (*p* < 0.05), as well as the supplementation with combined additives (11.5%). Combined additives showed an increase in egg masses, and additive association improved by 13.9% (*p* < 0.001) in contrast to the control treatment. Overall, β-mannanase and combined additives used during the supplementation period resulted in improvements in the weight of fresh eggs. These benefits were observed after a period of 14 weeks without supplementation (*p* < 0.05). Furthermore, significant differences were observed in the serum biochemistry and egg masses of birds that were fed diets containing both additives (β-mannanase + probiotics) compared to the control group. Parameters such as uric acid, total cholesterol, and triglycerides displayed notable variations. The villi height: crypt depth showed differences with combined additives (β-mannanase + probiotics). The β-mannanase improved specific gravity, yolk height, length, and pH, and yolk color traits compared to the control treatment. The use of probiotics helped to improve yolk height, pH, and color score. Besides, combined additives (β-mannanase + probiotics) improve yolk height, length, weight, pH, and better traits in yolk color. Hence, incorporating β-mannanase and probiotics into laying hen diets proves to be a highly effective strategy for enhancing laying rate and overall health status, while simultaneously elevating certain quality attributes of fresh eggs.

## 1 Introduction

In 2019, the World Health Organization (WHO) launched a campaign calling on governments to adopt measures to contain antimicrobial resistance. The inappropriate use of antibiotics both in human medicine and in animal production has become a public health problem, which has been worsening (1). The use of antibiotics is less frequent in laying poultry due to the possibility of residues in the eggs. Still, the use of feed additives is a possible alternative to improve productivity, health status, and even egg quality.

Probiotics were defined by Fuller (2) as a supplement consisting of live microorganisms that benefit the host and improve its intestinal microbial balance. The mechanisms of action of probiotics occur through different processes, which may or may not be associated. Physical effects arise from competitive exclusion or competition for binding sites on the intestinal mucosa. Beneficial bacteria present in probiotics form a protective physical barrier, preventing opportunistic pathogens from occupying the same binding sites. Biological effects occur due to the presence of anaerobic bacteria in probiotics. These bacteria promote a low oxygen tension environment within the gut, which inhibits the growth of pathogens, creating an unfavorable environment for their survival and reproduction. Chemical effects are observed through the production of bacteriocins by probiotic bacteria. Bacteriocins are antimicrobial substances that are effective against various pathogens, further inhibiting their growth and reducing their impact on the body. Additionally, probiotics can have a nutritional effect, providing essential nutrients and promoting a healthy gut environment, which supports the growth and maintenance of beneficial bacteria (3–6).

Enzyme supplementation is a valuable strategy for enhancing gut health by mitigating the effects of anti-nutritional components (7). β-mannanase, in particular, may support nonruminant animals in digesting non-starch polysaccharides, which can otherwise hamper nutrient digestibility (8–11). These polysaccharides, mainly β-mannans, are abundant in plant cell walls and commonly found in animal feed ingredients like soybeans (12). β-mannans are also present on the surface of microorganisms, triggering the animal’s innate immune system and leading to the activation of monocytes, macrophages, dendritic cells, and increased cytokine production. Consequently, this incurs unnecessary energy expenditure and heightened inflammatory response (13). By hydrolyzing β-mannans, this enzyme enhances mannans’ digestibility, boosts the population of beneficial bacteria, fortifies immunity, improves nutrient digestion and absorption, and restricts the proliferation of potential pathogens in the intestine (7).

Although the advantages mentioned earlier are noteworthy, it is important to note that the majority of the existing data was derived from different poultry categories, specifically broilers. Moreover, both additives function in complementary ways, suggesting the potential for synergistic effects when combined in feed supplementation. There is currently no literature available that describes the combined effects of these additives. Therefore, the objective of this study was to assess whether supplementing commercial laying hens with β-mannanase and probiotics alone or in combination could enhance performance, health status, and egg quality.

## 2 Materials and methods

### 2.1 Animals, housing, and experimental design

The experimental protocol described was approved by the Institutional Ethics Committee on the Use of Animals (CEUA/UFRGS) under protocol number 39783. The experimental units consisted of randomly selected hens from a commercial farm located in Salvador do Sul, Rio Grande do Sul, Brazil. The farm housed approximately 28,000 Hyline W 36 lineage light-weight laying hens, 36 weeks old. For the trial, 120 cages, each containing four birds, were utilized as replicates. These replicates were randomly assigned to the four treatments. The treatments consisted of: (1) Control (CON) treatment: This group received a basal diet without any supplementation with additional additives. (2) β-mannanase (BMA) treatment: The birds in this group received the control diet supplemented with 300 g/ton of β-mannanase. (3) Probiotic (PRO) treatment: The birds in this group received the control diet supplemented with 50 g/ton of a multi-strain probiotic additive. (4) β-mannanase + probiotic (BMA + PRO) treatment: The birds in this group received the control diet supplemented with 300 g/ton of β-mannanase and 50 g/ton of a multi-strain probiotic.

The β-mannanase employed in this trial was Hemicell™ HT, sourced from Elanco Animal Health in São Paulo, Brazil. It is an exogenous enzyme derived from the fermentation of the *Paenibacillus lentus* bacteria. The probiotic additive used in this trial was Protexin™ Concentrate, also provided by Elanco Animal Health in São Paulo, Brazil. The probiotic comprises a combination of beneficial bacterial strains, including: *Lactobacillus acidophilus* (2.06 × 108 UFC/g), *L. bulgaricus* (2.06 × 108 UFC/g), *L. plantarum* (1.26 × 108 UFC/g), *L. rhamnosus* (2.06 × 108 UFC/g), *Bifidobacterium bifidum* (2.0 × 108 UFC/g), *Enterococcus faecium* (6.46 × 108 UFC/g), and *Streptococcus thermophilus* (4.10 × 108 UFC/g).

The experiment spanned a duration of 26 weeks. During the initial 84 days of the trial, the birds received supplementation. To facilitate evaluation, this period was divided into three distinct phases: phase 1 (36–40 weeks), phase 2 (41–44 weeks), and phase 3 (45–48 weeks). Upon completion of the supplementation period, all birds were transitioned to the control diet for a period of 14 weeks. Subsequently, a new evaluation was conducted at week 62 to assess the outcomes.

The basal diet (Table 1) was formulated as a corn-soybean meal-based feed to fulfill the genetic nutritional requirements (14). To account for the absence of β-mannanase and/or probiotic additives, inert material in the form of kaolin was incorporated into the basal feed. During the entire experimental period, the birds had *ad libitum* access to feed and water. Nipple drinkers and gutter feeders were utilized, ensuring unrestricted access for the birds.

**Table 1:** Composition of control diet.

	Control treatment
Ingredient composition	
Corn	61.790
Soybean meal 45%	23.556
Limestone	9.283
Soybean oil	1.645
Dicalcium phosphate	1.549
Corn gluten 60%	1.024
Inert (washed sand)	0.262
Salt	0.497
DL-methionine	0.183
Vitamin premix^1^	0.100
Mineral Premix^2^	0.060
Choline chloride 70%	0.050
Calculated composition	
Metabolizable energy (kcal/kg)	2.800
Crude protein (%)	16.50
Calcium (%)	4.020
Available phosphorus (%)	0.380
Digestible methionine (%)	0.431
Digest. methionine+cystine (%)	0.668
Digestible lysine (%)	0.731
Digestible threonine (%)	0.559
Digestible tryptophan (%)	0.174
Digestible arginine (%)	0.984
Digestible valine (%)	0.690
Sodium (%)	0.220
Chlorine (%)	0.339
Potassium (%)	0.621

^1^Composition per kg of product: A vit.—10,000,000 IU; D3 vit.—2,500,000 IU; E vit.—6,000 IU; K vit.—1,600 mg; B12 vit.—11,000 mg; Niacin—25,000 mg; folic acid—400 mg; pantothenic acid—10,000 mg; and Se—300 mg. ^2^Composition per kg of product: MN—150,000 mg; zinc—100,000 mg; iron—100,000 mg; copper—16,000 mg; and iodine—1,500 mg.

The birds were accommodated in conventional sheds that were oriented in an east–west direction. These sheds were constructed with concrete floors and masonry walls, and wire mesh extended up to the ceiling. To ensure optimal thermal comfort, the sheds were equipped with adjustable side curtains, which were managed based on prevailing weather conditions. The lighting regime followed a schedule of 16 h of light starting at 04:00 pm until 08:00 am followed by 8 h of darkness each day, providing a consistent lighting pattern for the birds. Throughout the entire duration of the experiment, the birds were housed in galvanized-wire cages. These cages measured 100 cm in length, 40 cm in width, and 45 cm in height, offering a floor area of 500 cm^2^ per hen.

### 2.2 Performance analyses

Egg production was evaluated at weeks 4, 8, and 12 in 120 cages with four birds each, corresponding to 30 replicates per treatment. All eggs produced were individually weighed. Laying rate and egg mass were calculated considering all eggs (including non-marketable eggs) for each replicate (cage). The coefficient of variability was calculated for each cage considering the individual weight of all the eggs produced each week. The same procedure was adopted for egg masses [Egg mass = average weight (g) × percentage of egg production %]. Due to management limitations related to the commercial system, feed intake measurement was not possible in this study. For that reason, feed conversion was also not evaluated.

### 2.3 Dirtiness degree of the eggshells

All eggs produced in each repetition at weeks 4, 8, and 12 were individually inspected for the presence of feces in the shells, which was classified by the same observer through visual analysis as clean eggs (absent; score 0), minor presence (score 1), and major presence (score 3 and 4). During data analysis, scores 3 and 4 were considered together due to the low casuistry of score 3.

### 2.4 Serum biochemistry

At the end of week 12, blood samples were collected from eight randomly selected birds in each treatment. The samples were obtained from the ulnar vein of the birds, which were chosen from different cages. To obtain serum, the blood was collected in tubes without anticoagulant. The collected blood samples were then subjected to centrifugation at 3,500 rpm for 10 min. This process allowed for the separation of serum from other components of the blood. The resulting serum was carefully collected and frozen at −20°C to preserve its integrity for subsequent biochemical analysis.

For the biochemical analysis, the frozen serum samples were processed and examined using the Bio-Plus 2000® Biochemical Analyzer, manufactured by Bioplus in São Paulo, Brazil. Commercial kits from Wiener Lab Group, São Paulo, Brazil, were utilized to measure various parameters including total protein, albumin, uric acid, total cholesterol, triglycerides, glucose, alkaline phosphatase, alanine aminotransferase, and aspartate aminotransferase.

### 2.5 Parasitology test

At the conclusion of the experimental period, excreta samples were collected from three birds in each of the 10 cages. The collection was done with care, ensuring that the samples were processed within 2 h of collection to maintain their integrity. The centrifugal-flotation technique (15) was employed to process the excreta samples. To initiate the process, a subsample of 1 g of excreta was diluted in 15 mL of sucrose solution. This diluted sample was then subjected to centrifugation for 5 min. Following centrifugation, an optical microscope was utilized to examine the sample on a glass slide. The microscope was set at magnifications of 10, 40, and 100x to ensure accurate counting of oocysts.

### 2.6 Gut morphometric analyses

In accordance with the animal welfare and euthanasia standards outlined in the euthanasia practice guidelines of the National Council for Control of Animal Experimentation (16), six birds per treatment were humanely slaughtered using cervical dislocation. After euthanasia, 2-cm samples were collected from the duodenum, jejunum, and cecum of each bird. These tissue samples were carefully stored in flasks containing a 10% formaldehyde solution to preserve their structural integrity.

Histological slides were prepared from the collected tissue samples and stained with Archived Hematoxylin and Eosin (H&E). To capture the histological images, a microchamber Digital Eyepiece Camera Video, coupled with a biological trinocular microscope model TNB-41 T-PL at a magnification of 40x, was utilized. To determine the crypt depth and villus length, a line was measured from the base of the crypt to the upper portion for crypt depth. For villus length, a straight line was drawn from the tip of the villi to the upper portion of the crypts. ImageJ software bundled with 64-bit Java 1.8.0_172 was employed for accurate measurements.

### 2.7 Quality of fresh eggs

On the last day of weeks 4, 8, and 12, a total of 15 fresh eggs from each treatment in each phase were randomly collected for quality evaluation. Cracked eggs were excluded from this assessment. To determine the specific gravity, the eggs’ weight in both air and water was measured, following Archimedes’s principle.

The albumen height was determined by measuring three different points on the albumen, each 10 mm away from the yolk, using a digital caliper (TMX PD-150, China). The average of these measurements was used to calculate the Haugh Unit (HU), employing the equation developed by Haugh (17).

Yolk quality was evaluated by calculating the yolk index, which is the ratio of yolk height to yolk diameter, using the formula: YI = yolk height (mm)/yolk diameter (mm). Yolk height (mm) was measured using an altimeter, while yolk diameter (mm) was measured using a digital caliper.

Yolk width and height measurements (mm) were obtained using a digital caliper (TMX PD-150, China). The yolk index was calculated using the formula: yolk index = yolk weight/yolk width.

To assess yolk color, the Roche colorimetric fan (DSM, São Paulo, Brazil) was employed. This fan utilizes a scoring system ranging from 1 (representing a light yellow color) to 15 (indicating a reddish-orange hue). Additionally, a spectrophotometer device (Delta Vista model 450G, Delta Color, São Leopoldo, Brazil) was employed for this evaluation, providing colorimetric coordinates of luminosity (L*), red intensity (a*), and yellow intensity (b*). Chroma, which represents the actual yolk color for analysis, was estimated using the following equation: C = √(a^2^ + b^2^).

Once the yolk and albumen were separated, the weights of both components were measured. To ensure uniformity, the dense and fluid portions of the albumen, as well as the yolk, were homogenized for 20 s. To assess the pH levels, a digital pH meter (Kasvi model k39-2014B, Paraná, Brazil) was employed.

The total solid content of the albumen and yolk was assessed individually. For this, 5 g of albumen and yolk were weighed in pre-dried porcelain crucibles. The crucibles containing the samples were then placed in an oven set at 60°C for a duration of 12 h. Following the drying process, the crucibles were re-weighed to determine the weight of the dried albumen and yolk. To obtain the final solid content, the crucibles were subjected to a higher temperature of 105°C for another 12 h. After this drying step, the crucibles were weighed again to calculate the total solid content of the albumen and yolk accurately.

To determine the weight of the shell, a separate procedure was followed. The shells were carefully separated, thoroughly washed, and dried. Subsequently, the dried shells were weighed precisely to obtain their weight.

### 2.8 Statistical analyses

Data analyses were performed using the SAS statistical program (v 9.3, SAS Institute Inc., Cary, NC). Experimental units varied among the responses, but briefly, it was the cage for performance, the bird for biochemical and gut responses, and each egg for quality assessment. Data were tested for normality and homocedasticity and then submitted to variance analyses using PROC MIXED, except for the coefficient of variance of egg weight, which was analyzed using PROC GLIMMIX. All statistical models included the fixed effect of treatments and the error. Performance data were analyzed considering repeated measures over time. Egg quality was analyzed considering also the random effect of phase in the model, although only pooled means are presented here due to the lack of interaction between treatment and phase. Eventual mean differences were compared by the Tukey test at 5 and 10% probability.

## 3 Results

The measurements of temperature and humidity were obtained using a datalogger. The average recorded values for the minimum and maximum temperatures were 18 and 36°C, respectively. The average air relative humidity values ranged from 35.8 to 94.7%. This study was conducted in Salvador do Sul (Southern region, Brazil) which largely experiences a subtropical climate. The project implementation started in December (summer) and lasted until March (summer-autumn). Throughout the entire trial, the animals exhibited performance consistent with the expectations for their specific genotype. Furthermore, the animals remained in good health throughout the experimental period, as no severe health problems or illnesses were observed.

### 3.1 Performance and dirtiness degree of the eggshells

In phase 1, the BMA and BMA + PRO groups presented a 12% higher posture rate compared to the CON (*p* < 0.001, Table 2). In phase 2, all supplemented treatments had higher laying rates (21%) compared to CON (*p* < 0.001). In phase 3, the group fed with BMA + PRO was 5% superior to CON (*p* < 0.001), while the BMA and PRO groups were intermediate in relation to CON and BMA + PRO. In the overall period, all treatments had a higher (*p* < 0.001) laying rate compared to CON, in which BMA + PRO had the highest laying rate followed by BMA and PRO.

**Table 2:** Performance of laying hens fed diets supplemented with β-mannanase (BMA) and/or probiotics (PRO).

Responses	Treatments				SEM^1^	*p* value^2^
	CON	BMA	PRO	BMA + PRO		
Laying rate (%)						
Phase 1^4^	85.31^C^	93.71^B^	83.25^C^	97.66 ^A^	0.11	<0.001
Phase 2	78.34 ^B^	96.69 ^A^	96.36 ^A^	90.55 ^A^	0.11	<0.001
Phase 3	91.05 ^B^	92.59 ^AB^	92.60 ^AB^	95.90 ^A^	0.09	<0.001
Overall	84.90 ^C^	94.33 ^A^	90.74 ^B^	94.70 ^A^	0.59	<0.001
Weight of fresh eggs (g)						
Phase 1	61.95 ^B^	62.36 ^B^	61.38 ^B^	63.18 ^A^	0.14	<0.001
Phase 2	61.28 ^C^	63.09 ^A^	61.98 ^B^	62.79 ^A^	0.15	<0.001
Phase 3	64.13 ^B^	65.21 ^A^	63.76 ^B^	65.52 ^A^	0.16	<0.001
Overall	62.47 ^B^	63.55 ^A^	62.37 ^B^	63.83 ^A^	0.09	<0.001
62 weeks^3^	62.53 ^B^	64.40 ^A^	64.10 ^A^	64.28 ^A^	0.13	0.013
Coefficient of variability in egg weight (%)						
Phase 1	5.944 ^b^	5.897 ^b^	5.193 ^a^	5.292 ^ab^	0.014	0.072
Phase 2	7.152 ^B^	5.625 ^A^	5.750 ^A^	5.734 ^A^	0.018	0.007
Phase 3	7.088 ^B^	5.397 ^A^	5.608 ^A^	5.405 ^A^	0.019	0.004
Overall	6.728 ^B^	5.640 ^A^	5.517 ^A^	5.477 ^A^	0.01	<0.001
62 weeks^3^	7.94	8.285	8.272	7.891	0.124	0.564
Total solids^5^						
Albumen	10.78	10.59	10.66	11.59	0.21	0.383
Yolk	50.28	49.94	48.73	50.51	0.38	0.339

^1^Standart error of mean. ^2^Probability of treatment effect. Means followed by different uppercase letters differ statistically at 5%, while lowercase letters were used to indicate differences at 10%. ^3^Treatments were not provided from week 48 to 62. Thus, the last evaluation was performed after 14 weeks without supplementation. ^4^Phase 1: 36–40 weeks; phase 2: 41–44 weeks; and phase 3: 45–48 weeks.

Regarding egg weight, the BMA + PRO group differed from the CON, with higher (*p* < 0.001) egg weight in phase 1. In phase 2, all treatments differed (*p* < 0.001) from CON, with BMA and BMA + PRO being similar to each other. In phase 3, the PRO and BMA + PRO treatments differed from CON (*p* < 0.001); however, the PRO group was similar to the control which also occurs in the overall period. At week 62, all treatments differed (*p* = 0.013) from CON.

All treatments differed (*p* < 0.001) from CON in terms of the overall coefficient of variability of egg weight, with the lowest values observed in BMA + PRO group. A trend effect (*p* = 0.072) was observed in phase 1, with the lowest coefficient of variability attributed to PRO treatment. In phases 2 (*p* = 0.007) and 3 (*p* = 0.004) all treatments differed from CON, with lowest values observed in BMA group. However, at week 62 (after treatment removal), no significant differences were observed among the groups (*p* = 0.564).

Regarding egg masses (Table 3), in phases 1 and 3 (*p* < 0.001), treatments BMA and BMA + PRO were different from the CON group, showing higher egg masses. In phase 2 and overall (*p* < 0.001), all treatments increased egg masses compared to the CON.

**Table 3:** Egg masses of laying hens fed diets supplemented with β-mannanase (BMA) and/or probiotics (PRO).

Responses	Treatments				SEM^1^	*p* value^2^
	CON	BMA	PRO	BMA + PRO		
Egg mass (g/hen/day)						
Phase 1^3^	52.85 ^B^	58.44 ^A^	51.10 ^B^	61.70 ^A^	0.67	<0.001
Phase 2	48.01 ^C^	61.00 ^A^	59.72 ^AB^	56.86 ^B^	0.39	<0.001
Phase 3	58.30 ^B^	60.38 ^A^	59.04 ^AB^	62.83 ^A^	0.63	<0.001
Overall	53.08 ^C^	59.94 ^A^	56.62 ^B^	60.46 ^A^	0.39	<0.001

^1^Standart error of mean. ^2^Probability of treatment effect. Means followed by different uppercase letters differ statistically at 5%, while lowercase letters were used to indicate differences at 10%. ^3^Phase 1: 36–40 weeks; phase 2: 41–44 weeks; phase 3: 45–48 weeks.

**Figure 1:**
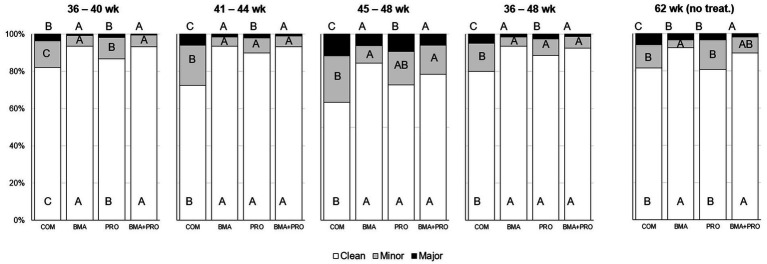
Occurrence of clean eggs or minor/major presence of feces (%) in eggs from laying hens fed β-mannanase and/or probiotics. Comparisons were performed among treatments in each period. The probability of treatment effect was *p* < 0.001 for all responses, except for the period from 45 to 48 weeks in which all responses showed *p* < 0.05. Different uppercase letters differ statistically at 5%. Treatments were not provided from week 48 to 62. Thus, the last evaluation was performed after 14 weeks without supplementation.

The occurrence of clean eggs in treated birds differed from CON in all phases of the experiment (*p* < 0.05, Figure 1). The BMA, PRO, and BMA + PRO groups were superior to CON in all phases. In the 62nd week of production, after 14 weeks without supplementation, the occurrence of clean eggs still differed (*p* < 0.001) in the BMA and BMA + PRO groups compared to CON.

### 3.2 Serum biochemistry

Uric acid differed from CON (*p* < 0.001) in all treatments, with the lowest values observed in BMA + PRO, BMA, and PRO, respectively (Table 4). Total cholesterol and triglycerides differed from CON (*p* < 0.001), with smaller values shown in supplemented treatments. Serum glucose levels were higher in CON (*p* < 0.001) birds in relation to the BMA and BMA + PRO treatments. The PRO treatment did not differ from the CON. Serum alkaline phosphatase was higher (*p* = 0.007) in PRO and BMA + PRO treatments than in CON. The BMA was similar to CON and other treatments in this study. Alanine aminotransferase was higher in CON (*p* = 0.005) than in PRO and BMA + PRO treatments.

**Table 4:** Serum biochemistry and intestinal morphometry of laying hens fed β-mannanase and/or probiotics.

Responses	Treatments				SEM^1^	*p* value^2^
	CON	BMA	PRO	BMA + PRO		
Serum biochemistry						
Total protein (g/dL)	5.475	7.113	5.038	6.575	0.361	0.148
Albumin (g/dL)	1.850	2.100	1.875	2.043	0.052	0.237
Uric acid (mg/dL)	5.171 ^A^	2.400 ^BC^	3.171 ^B^	2.062 ^C^	0.247	<0.001
Total cholesterol (mg/dL)	301.3 ^A^	149.5 ^B^	204.1 ^B^	161.9 ^B^	14.1	<0.001
Triglycerides (mg/dL)	832.1 ^A^	929.0 ^A^	539.0 ^B^	659.6 ^B^	45.6	0.013
Glucose (mg/dL)	367.0 ^A^	293.0 ^B^	401.5 ^A^	269.1 ^B^	11.5	<0.001
FA (U/L)^3^	378.1 ^B^	624.0 ^AB^	896.0 ^A^	831.8 ^A^	64.0	0.007
ALT (U/L)	8.207 ^A^	7.020 ^A^	3.201 ^B^	1.667 ^B^	0.811	0.005
AST (U/L)	144.3	149.7	149.0	140.4	2.7	0.579
Gut morphometry						
Villi height (μm)	1,459	1,294	1,375	1,561	30.6	0.428
Villi width (μm)	249.0 ^ab^	226.7 ^b^	246.4 ^ab^	283.1 ^a^	5.69	0.064
Villi area (μm^2^)	367,039	288,838	343,272	450,579	1,798	0.67
Crypt depth (μm)	224.9	186.4	227.6	202.1	4.96	0.397
Villi height: Crypt depth	6.839 ^B^	7.245 ^AB^	6.304 ^B^	8.396 ^A^	0.171	0.007

^1^Standart error of mean. ^2^Probability of treatment effect. Means followed by different uppercase letters differ statistically at 5%, while lowercase letters were used to indicate differences at 10%. ^3^FA, Alkaline phosphatase; ALT, Alanine aminotransferase; AST, Aspartate aminotransferase.

No significant differences were observed in aspartate aminotransferase (*p* = 0.579) and total protein (*p* = 0.148). There were also no significant differences in serum albumin (*p* = 0.237).

### 3.3 Gut morphometry and parasitological analysis

No difference was observed in villus height, villus area, and crypt depth among the treatments. The villi width tended (*p* = 0.064) to be smaller in the BMA treatment compared to the control, whereas the BMA + CON treatment tended to be superior to CON and the PRO treatment was similar to CON. The relationship between the height of the villus and the depth of the crypt was significant (*p* = 0.007), with the highest relationship observed in the BMA + PRO treatment compared to the CON. The PRO treatment was similar to CON and BMA similar to CON and BMA + PRO.

No parasites or oocysts were found in the fresh excrete samples, including the control treatment. This condition did not allow the evaluation of the eventual effect of treatments on parasite challenges.

### 3.4 Quality of fresh eggs

The BMA group showed higher (*p* < 0.001) specific gravity when compared to the CON (Table 5), with higher values. In addition, higher (*p* = 0.009) shell weights were observed in the BMA and BMA + PRO groups compared to CON.

**Table 5:** Quality of fresh eggs from laying hens fed diets supplemented with β-mannanase (BMA) and/or probiotics (PRO).

Responses^3^	Treatments				SEM^1^	*p* value^2^
	CON	BMA	PRO	BMA + PRO		
General traits						
Spec. gravity (g/ml)	1.006 ^B^	1.007 ^A^	1.006 ^B^	1.006 ^B^	0.001	<0.001
Albumen traits						
Height (mm)	8.04	8.06	8.18	8.17	0.104	0.129
Weight (g)	36.82	37.39	36.30	36.59	0.239	0.424
pH	8.41	8.40	8.38	8.44	0.028	0.178
Yolk traits						
Height (mm)	17.98 ^B^	18.15 ^A^	18.27 ^A^	18.18 ^A^	0.063	0.037
Length (mm)	40.67 ^B^	41.62 ^A^	41.25 ^AB^	41.82 ^A^	0.118	0.002
Index	0.443	0.435	0.443	0.435	0.017	0.194
Weight (g)	15.33 ^B^	15.70 ^AB^	15.45 ^B^	16.08 ^A^	0.096	0.004
Haugh unit	89.40	90.10	89.88	89.55	0.558	0.132
pH	6.04 ^B^	5.96 ^A^	5.99 ^A^	6.00 ^A^	0.013	0.002
Yolk color						
Color score	5.60 ^B^	5.98 ^A^	5.77 ^AB^	5.87 ^AB^	0.052	0.032
Lightness (L*)	50.85 ^B^	50.66 ^B^	51.33 ^AB^	52.16 ^A^	0.161	0.002
Redness (a*)	7.12 ^B^	7.66 ^A^	7.67 ^A^	7.66 ^A^	0.100	<0.001
Yellowness (b*)	57.41	58.88	58.93	58.75	0.354	0.122
Chroma	57.85 ^B^	59.67 ^A^	59.67 ^A^	59.25 ^A^	0.357	0.003
Shell traits						
Weight (g)	5.81 ^B^	6.15 ^A^	5.96 ^AB^	6.11 ^A^	0.041	0.009

^1^Standart error of mean. ^2^Probability of treatment effect. Means followed by different uppercase letters differ statistically at 5%, while lowercase letters were used to indicate differences at 10%. ^3^A subsample of 15 eggs from each treatment in each phase.

Yolk height showed significant differences in the BMA, PRO, and BMA + PRO groups compared to CON (*p* = 0.037), all with higher values. Yolk width was also higher (*p* = 0.002) in the BMA and BMA + PRO groups compared to CON. Yolk weight was higher in the BMA + PRO group (*p* = 0.004) compared to the CON group. Yolk pH, on the other hand, differed from CON in all groups (*p* = 0.002), showing lower pH values.

**Figure 2:**
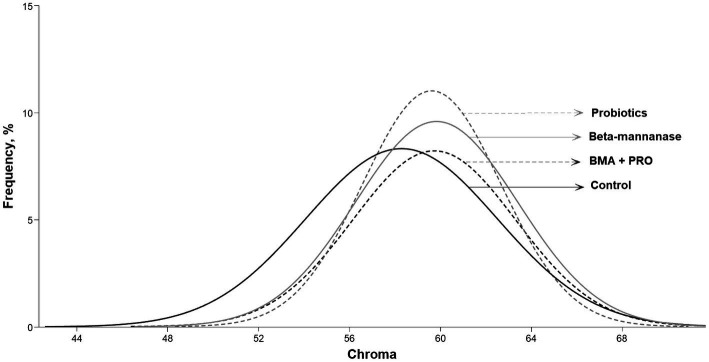
Frequency of different chroma indexes in egg yolks from laying hens fed β-mannanase and/or probiotics.

The BMA group showed a higher color score in the Roche colorimetric assessment compared to the CON (*p* = 0.032). Regarding luminosity values (L* color), the BMA + PRO group was superior to the CON group (*p* = 0.002). Such findings indicate lower luminosity, that is, they were opaquer as they transmit less light. Higher red intensity (A* color) and chroma values were observed in all supplemented groups than CON (*p* < 0.001; Figure 2).

## 4 Discussion

### 4.1 Performance and dirtiness degree of the eggshells

In the current study, an improvement in overall laying rates across all treatments were observed when compared to the control group. This finding aligns with previous research conducted by Zhan et al. (18), who reported a significant increase in laying rates in birds when fed with probiotics, particularly when administered at a dosage of 5 × 104 cfu/g of *Clostridium butiricum*. Similarly, Ribeiro et al. (19) documented a substantial increase in laying rates with a dosage of 8 × 105 cfu/g of *Bacillus subtilis*, while Saleh et al. (20) observed improvement when utilizing 0.05% of *Aspergillus awamori*. Probiotics have been recognized for their positive impact on egg production, which is attributed to several beneficial mechanisms. These include enhanced nutrient absorption (19), improved immune function, reduced stress in birds, and promotion of intestinal health (18) which are effects that may help explain the results found in this study. In this context, it is important to mention that the current study was developed under commercial farm conditions. Such condition generally indicates that animals are exposed to certain challenges (biosecurity, environment, number of animals, among others) that would be limited under small-scale experimental conditions. Both study types are relevant and able to provide useful data. However, this characteristic needs to be considered when interpreting the results.

In reference to β-mannanase, our current findings show an enhanced laying rate when compared to the control treatment. These results are in agreement with the results of Zheng et al. (21), who reported a significant boost in egg production among laying hens that were supplemented with β-mannanase on low-energy diets. Notably, the values were similar to high-energy diets without enzymes. Similar data were also found by Wu et al. (22). These findings may be associated with the fact that β-mannanase, by avoiding the immune response triggered by β-mannanase, redirects energy and nutrients for the bird’s performance (23).

The current study noted a significant increase in egg weight, aligning with previous research by Khan et al. (24), Alaquil et al. (25), and Mikulski et al. (26) in diets supplemented with probiotics. Moreover, concerning β-mannanase, our findings agree with the results of Ryu et al. (27), when using 0.8 g β-mannanase/kg, observed an increase in egg weight compared to the control. Regarding the coefficient of variability of egg weight, we observed a stable and predictable production, which facilitates the processes and increases profitability by decreasing the number of declassified eggs.

In regard to egg masses, our study revealed an increase in egg masses across all treatments when compared to the control. These results agree with Ryu et al. (27) that observed higher egg masses using β-mannanase. This effect may be attributed to the improved digestion and utilization of dietary components, leading to increased nutrient absorption and utilization for egg production. As for probiotics, Saleh et al. (20), Alaquil et al. (25), and Ribeiro et al. (19) also observed higher egg masses when compared to the control group. Probiotics are known to foster a balanced gut microbiota, which can positively influence nutrient metabolism and absorption, subsequently contributing to improved egg mass production.

The higher occurrence of clean eggs in treated birds observed in this study differed from the control in all phases of the experiment. The use of β-mannanase can be explained by the probable decrease in feces viscosity. Soluble non-starch polysaccharides increase digesta viscosity by increasing water retention, impairing nutrient diffusion and transport. Daskiran et al. (28) demonstrated that diets that used β-mannanase significantly reduced the water of total fecal production in broilers. Likewise, Mehri et al. (29) demonstrated that the viscosity of digesta from the jejunum of broiler chickens decreased in diets with the enzyme. The cleaner eggs observed in the probiotic treatment can likely be attributed to the enhanced stability of the intestinal microbiota and lower count of opportunistic bacteria. Higgins et al. (30) and Deng et al. (31) observed a decrease in *Salmonella* sp. colonies in birds supplemented with probiotics. Aalaei et al. (32) found that the addition of multi-strain probiotics reduced the presence of *E. coli* in broilers, and with that, it was possible to reduce diarrhea in the birds. Therefore, in the present study, the reduction in diarrhea could be the cause of the decrease in dirty eggs from the PRO treatment. To our knowledge, this is the first study that evaluates the occurrence of dirty eggs in laying hens fed β-mannanase alone or combined with probiotics.

### 4.2 Serum biochemistry

Uric acid is the main product of nitrogen metabolism in birds, which is synthesized in the liver and kidneys. Disorders in renal function can increase the concentration of uric acid in the serum and plasma of birds (33), as well as elevated temperatures (34). Low uric acid levels also indicate lower protein turnover (35), that is, lower endogenous losses of nitrogen and ammonia. In the present study, which took place in summer, the birds faced high temperatures, and even so the values of uric acid found in the blood were lower in all treatments compared to the control, which may indicate an improvement in the health of the birds and better efficiency in protein utilization due to additives.

Furthermore, in relation to protein metabolism, uric acid may elucidate the observed phenomena of increased albumen weight and egg mass in this study, potentially attributable to enhanced protein deposition within these eggs.

Total cholesterol and triglyceride levels were significantly lower in the groups that received probiotics and the combined additives (BMA + PRO) when compared to the control group. These findings are consistent with previous research (20, 36, 37) in birds that were supplemented with probiotics. Furthermore, birds that were fed β-mannanase exhibited lower total cholesterol levels, which aligns with the results reported by Karimi and Shokrollari (38), who observed a decrease in LDL-cholesterol levels. Serum cholesterol and triglyceride levels reflect lipid metabolism. Saleh et al. (39) reported that one of the possible mechanisms of cholesterol reduction by probiotics occurs through the production of HMG-CoA reductase (3-hydroxy-3-methyl-glutaryl-CoA reductase), which reduces the deposition of abdominal fat by influencing the activity of the hormone-sensitive lipase and malate dehydrogenase enzyme in adipose tissues (40). Also, one of the supposed mechanisms of probiotics occurs through the reduction of hepatic bile acid synthesis (41). Lactic acid bacteria such as those found in the tested product have the ability to reduce cholesterol in the bloodstream (42). The decrease in cholesterol by β-mannanase can be explained by the hypolipidemic effect of the enzyme, which reduces the absorption of lipids (38, 43).

Regarding serum glucose, our findings are in agreement with Tang et al. (44). The decrease in glucose by β-mannanase can be explained by the fact that this enzyme stimulates insulin secretion (45), which may stimulate feed intake behavior and consequently be linked to increasing egg production.

The increase in serum alkaline phosphatase agrees with the findings of Yalcin et al. (37). And the higher alanine aminotransferase also is in agreement with Saleh et al. (39) and Tang et al. (44). The serum concentration of liver enzymes such as alkaline phosphatase and alanine aminotransferase can provide information about tissue and organ damage (46). Alkaline phosphatase is also associated with calcium and phosphorus metabolism and with participation in osteoblastic and chondrogenic activities. Therefore, the increase in this enzyme is associated with bone growth, fracture consolidation, and pre-ovulation and medullary calcification phase in chickens (33). Furthermore, changes in alkaline phosphatase levels may indicate that the medullary bone promotes calcium during the formation of eggshells and stores calcium when there is no egg in the uterus (46). In relation to alanine aminotransferase in birds, it is believed that it may be elevated due to damage to multiple tissues, making its interpretation difficult (47). In the present study, results observed that the birds fed with PRO, and BMA + PRO groups had higher serum alkaline phosphatase values, which indicates better health for these birds. The lower values of alanine aminotransferase observed in this study may indicate a more efficient metabolism of these birds due to less liver damage, which may explain the positive performance results.

### 4.3 Gut morphometry and parasitological analysis

Crypt height and depth measurements are often used to assess intestinal integrity. The height of the villi indicates a greater area for nutrient absorption and a deeper crypt indicates that there is greater tissue renewal (48, 49). In the present study, the group treated with BMA + PRO showed a greater villus height: crypt depth ratio, demonstrating an improvement in intestinal health.

Previous studies have shown significant differences in the ratio between villus height and crypt depth (36) in the intestine of laying hens fed with probiotics. Even though there are no studies in relation to β-mannanase in laying hens, based on these results, it is believed that this additive can benefit the intestinal health of birds. The higher villus: crypt ratio in these groups, as it is associated with a greater surface area for nutrient absorption, may explain the better performance of these birds, especially in relation to egg weight and egg mass.

### 4.4 Quality of fresh eggs

It is crucial to recognize that the composition of the eggshell primarily consists of calcium carbonate, along with magnesium carbonate and calcium phosphate, among other components. The balance between calcium and phosphorus ions plays a vital role in the formation of the eggshell (50). Specific gravity is an indicator of the proportion of the shell in relation to other components of the egg. It is closely associated with shell thickness and, consequently, the deposition of calcium carbonate. Evaluating the specific gravity provides insights into the quality of the shell. Additionally, shell weight can be used to support the findings obtained from specific gravity measurements and assess calcium metabolism.

In our study, we observed higher specific gravity and increased eggshell weight in the BMA group compared to the CON. These findings suggest a higher quality of the shell and a reduced likelihood of breakage during handling (51, 52). The improved specific gravity and increased eggshell weight indicate enhanced shell integrity and strength, which are desirable attributes in terms of egg quality and marketability.

Yolk and albumen weight exhibit a positive correlation with egg weight. Eggs with higher weights tend to have greater yolk and albumen masses compared to those with lower weights. Egg weight is influenced by various factors, including heritability, age, and bird weight (53). Additionally, egg weight strongly influences dietary protein requirements (54). In this study, we observed lower uric acid levels in birds fed with the BMA group compared to the CON group, suggesting reduced protein turnover. β-mannans, found in the diet, are known to reduce viscosity and inhibit enzyme action (55). The addition of β-mannanase in the BMA group facilitated enzyme activity by breaking down β-mannans, potentially leading to increased protein absorption. This mechanism may explain the higher yolk weight observed in the BMA group. Another hypothesis is associated with the viscosity-reducing effect of β-mannanase (56). This enzymatic activity alters the structure of micelles (57, 58), which are lipid compounds of significant importance as they are deposited in the yolk. The decrease in viscosity caused by β-mannanase could potentially enhance the formation or function of micelles, contributing to an increased yolk weight.

The results related to yolk color demonstrated lower luminosity in the BMA + PRO group compared to the CON, indicating a more opaque appearance and reduced light transmission. Additionally, the PRO and BMA groups exhibited desirable yellowish and reddish colors, which are considered attractive to consumers (59). The pigmentation of the yolk occurs through the absorption of carotenoid pigments present in the bird’s diet (60). Corn, for instance, contains carotenoids such as xanthophyll, lutein, and zeaxanthin (61). These lipophilic and unsaturated compounds accumulate in the yolk, which has the highest concentration of fat in the egg (62). One hypothesis for the observed color changes is that β-mannanase may enhance nutrient absorption and/or increase the production of micelles, which are responsible for transporting carotenoids and accumulating them in the yolk. However, further studies are needed to validate this hypothesis.

It is worth mentioning that parameters such as yolk pH, height, and width are indicators of egg freshness (63, 64). In this trial, the treatments resulted in decreased pH values and increased yolk height and width, indicating improved egg quality and freshness.

## 5 Conclusion

The present study provides evidence that the supplementation of β-mannanase, probiotics, and their combination in feed can significantly enhance performance (laying rate and weight of fresh eggs) and gut health (ratio villi height to crypt depth). Furthermore, the supplementation of these additives in the poultry feed leads to the occurrence of eggs more homogeneous in terms of weight, cleaner, and with an better quality.

## Data availability statement

The original contributions presented in the study are included in the article/supplementary material, further inquiries can be directed to the corresponding author.

## Ethics statement

The animal study was approved by the experimental protocol described was approved by the Institutional Ethics Committee on the Use of Animals (CEUA/UFRGS) under protocol number 39783. The study was conducted in accordance with the local legislation and institutional requirements.

## Author contributions

CC, IA, and MK: conceptualization, methodology, validation, and formal analysis. CC, GG, NC, TB, RiM, and GP: investigation. IA and MK: resources, visualization, project administration, and funding acquisition. CC and IA: data curation. CC: writing—original draft preparation. CC, IA, GG, BB, and MK: writing—review and editing. IA, RaM, and MK: supervision. All authors contributed to the article and approved the submitted version.
